# Rapid Iodine Sensing on Mechanically Treated Carbon Nanofibers

**DOI:** 10.3390/s18051486

**Published:** 2018-05-09

**Authors:** Eunbyul Cho, Alexandra Perebikovsky, Olivia Benice, Sunshine Holmberg, Marc Madou, Maziar Ghazinejad

**Affiliations:** 1 Department of Biomedical Engineering, University of California, Irvine, CA 92697, USA; eunbyuc@uci.edu; 2 Department of Physics and Astronomy, University of California, Irvine, CA 92697, USA; aperebik@uci.edu; 3 Department of Chemistry, University of California, Irvine, CA 92697, USA; obenice@uci.edu; 4 Department of Mechanical and Aerospace Engineering, University of California, Irvine, CA 92697, USA; sholmber@uci.edu (S.H.); mmadou@uci.edu (M.M.); 5 Department of Mechanical Engineering, California State University, Fresno, CA 93740, USA

**Keywords:** carbon nanofiber, electrochemical sensor, iodine deficiency, carbon microstructure, point-of-care

## Abstract

In this work, we report on a rapid, efficient electrochemical iodine sensor based on mechanically treated carbon nanofiber (MCNF) electrodes. The electrode’s highly graphitic content, unique microstructure, and the presence of nitrogen heteroatoms in its atomic lattice contribute to increased heterogeneous electron transfer and improved kinetics compared to conventional pyrolytic carbons. The electrode demonstrates selectivity for iodide ions in the presence of both interfering agents and high salt concentrations. The sensor exhibits clinically relevant limits of detection of 0.59 µM and 1.41 µM, in 1X PBS and synthetic urine, respectively, and a wide dynamic range between 5 µM and 700 µM. These results illustrate the advantages of the material’s unique electrochemical properties for iodide sensing, in addition to its simple, inexpensive fabrication. The reported iodine sensor eliminates the need for specimen processing, revealing its aptitude for applications in point-of-care diagnostics.

## 1. Introduction

Iodine is an essential micronutrient that plays a key role in thyroid hormone synthesis and brain development. Iodine deficiency disorder (IDD) is a major world health problem that leads to increased perinatal mortality, birth defects, hypothyroidism and a host of other functional and developmental abnormalities [1]. For example, if iodine deficiency occurs during the most critical period of fetal brain development, the resulting thyroid failure leads to irreversible alterations in brain function [2].

One of the most critical aspects to controlling IDD is accurate and inexpensive monitoring of iodine concentration in physiological fluids. Since excess iodine is secreted in the urine of individuals, urinary iodine concentration is the main epidemiological indicator of IDD, with levels below 100 μg/L classified as deficient in healthy adults. During pregnancy, where IDD-induced neurological defects in the fetus are more common, optimal UI concentration is defined between 150–250 μg/L [3].

While many methods have been developed to detect trace quantities of iodine, they are outdated, often expensive, and involve complex laboratory handling methods and instrumentation [4].

Commercially, urinary iodide concentration is exclusively measured colorimetrically by the Sandell-Kolthoff method, in which yellow cerium (IV) is reduced to cerium (III) in the presence of iodide ions. This method requires complicated sample preparation, involves a slow response time, and utilizes a toxic reagent that must be properly stored and disposed [5].

The goal in iodine monitoring research is to develop simple and sensitive methods for detection of iodine by using cost-effective probes that require no extra reagents or sample preparation. Ideally, these methods should have fast response times, low limits of detection, a wide dynamic range, and capability of detecting iodide ions in the presence of interfering substances. Various nanomaterials, such as ZnO nanotubes-functionalized gold-coated glass, poly(3,4-ethylenedioxythiophene)/glassy carbon composite, plasticized polyvinyl chloride with two fluorescence, graphene quantum dots/silver nanocomposites, have been used for developing iodide sensors [6,7,8,9]. However, these sensing materials often use expensive precious metals such as gold or silver and require additional post fabrication chemical treatments or expensive optical setups to be effective sensors.

A promising solution is to use electrochemical methods that take advantage of the electroactivity of iodide without adding any pretreatment steps. Silver working electrodes are commonly used for these electrochemical methods, exhibiting detection limits down to 40 nM and linear ranges from 0.2 to 1.6 µM [10]. However, silver electrodes need to be replaced frequently, since they operate by forming a silver iodide (AgI) precipitate on the surface. This issue is also present in expensive platinum (Pt) or gold (Au) electrodes, where the detection of concentrations greater than or equal to 1 mM results in surface passivation of the electrode [11,12].

An alternative approach is the development of iodide-sensitive electrodes using inexpensive carbon materials, such as glassy carbon and pyrolytic carbon fibers, which are chemically stable and exhibit a wide potential window. However, carbon electrodes often require post-synthesis processing (e.g., surface modification or inserting heteroatoms) to become effective iodine sensors. These methods can be complex, and, in the case of iodine, the resulting processes are transient. In one of the few related studies, Lowe et al. were able to detect low micromolar concentrations of iodide in a 0.1 mM sulfuric acid solution by exploiting the adsorption of iodide ions onto edge-plane pyrolytic graphite electrodes [13].

In this work, we demonstrate the detection of clinically relevant concentrations of iodide in synthetic urine and in the presence of interfering ions by using mechanically-treated pyrolytic carbon nanofiber (MCNF) electrodes. The unique microstructure of MCNF electrodes significantly improves its heterogeneous electron transfer rate [14], rendering them efficient, electrochemically active sensors. The MCNF material was used directly as an iodide selective electrode, without any post-processing or secondary activation methods. Cyclic voltammetry (CV) and differential pulse voltammetry (DPV) were used to characterize its efficacy as an iodine sensor. The enhanced electrochemical kinetics of MCNF electrodes in urine allow them to efficiently detect iodide ions and distinguish them from other interfering redox species that are present in synthetic urine. Based on the DPV characterization, MCNF electrodes exhibit 0.59 µM and 1.41 µM limits of iodine detection in 1X PBS and urine solutions, respectively. The results of this study demonstrate the promising capability of MCNF sensors for rapid, qualitative detection and monitoring of iodine in urine specimens.

## 2. Materials and Methods

### 2.1. Materials and Instruments

All chemicals used were of reagent grade. Multi-walled carbon nanotubes (MWCNT) and polyacrylonitrile (PAN, M.W. = 150,000 u) were purchased from Sigma Aldrich, St. Louis, MO, USA. *N*, *N*-dimethylformamide 99.9% (DMF), potassium iodide (KI), and phosphate buffered saline (PBS) tablets were obtained from Fisher Scientific, Hampton, NH, USA. Artificial urine, pH 6.6, compliant with DIN EN 1616:1999, was purchased from Pickering Laboratories, Mountain View, CA, USA.

### 2.2. Synthesis of Mechanically-Treated Carbon Nanofiber (MCNF) Electrodes

Polyacrylonitrile (PAN, M.W. = 150,000 g/mol) was mixed with *N*,*N*-dimethylformamide (DMF 99.9%, Fisher Chemical) and multi-walled carbon nanotubes (MWCNT) to produce 8% (*v*/*v*) PAN and 1% (*w*/*v*) MWCNT-DMF solution. This precursor solution was spun by a far-field electrospinning technique using 12 kV electrical potential and 0.9 mL/h flow rate. The PAN-CNT nanofiber mats were then mechanically rolled using a Dayton’s DC Speed Control Roller. The resulting mat was stabilized at 280 °C in air, and pyrolyzed at 1000 °C under nitrogen flow, to produce the mechanically treated carbon nanofibers (MCNF).

**Figure 1:**
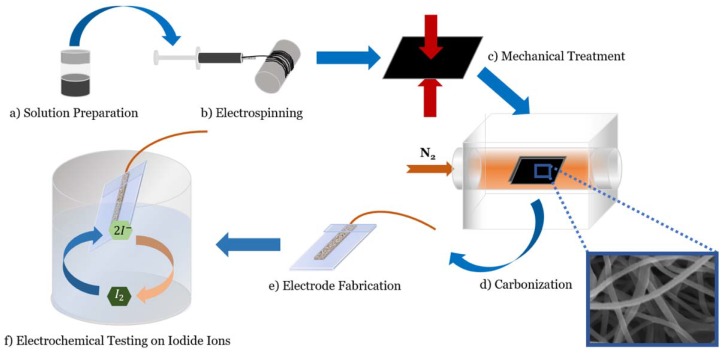
Schematiciagram of the fabrication and electrochemical testing of the MCNF electrodes. (**a**,**b**) PAN-CNT solution is electrospun onto a rotating drum via a far-field electrospinning technique. (**c**) the resulting nanofibers are treated with compressive, mechanical stress; (**d**) mechanically-treated carbon nanofibers (MCNF) are carbonized at 1000 °C under nitrogen flow; (**e**,**f**) MCNF’s are cut into electrodes and electrochemically tested in 1X PBS buffer and synthetic urine with different iodide concentrations.

As described comprehensively in our previous report, the addition of MWCNT to PAN DMF solution introduces dielectrophoretic forces in the electrospinning process, and results in velocity gradients at the interface of the MWCNTs and polymer chains. This phenomenon produces localized shear stress fields at the surface of MWCNTs, thus unwinding and aligning PAN molecular chains within these zones. Upon pyrolysis, a unique graphitic microstructure is formed, which is rich in carbon edge planes and nitrogen heteroatoms. It is important to note that the presence of these edge planes is critical to the electrochemical performance of carbon materials. While CNTs have a graphitic microstructure, it is mostly comprised of basal planes, and therefore is not as electrochemically active as “fragmented” graphitic structures, which contain a higher quantity of electroactive sites. Accordingly, the role of MWCNT is to align PAN precursor molecular chains that, in combination with applied mechanical stresses during the stabilization, will generate a fragmented graphitic microstructure with enhanced electrochemical response. The MCNF material was directly used to fabricate carbon electrodes with no additional post-synthesis processing. Figure 1 summarizes the fabrication process for MCNF electrodes.

### 2.3. Electrochemical Characterization

Electrochemical measurements were conducted using a Princeton Applied Research VersaSTAT 4 Potentiostat running VersaStudio 2.48.5 software. All electrochemical experiments were run in 1X PBS solution, which was made by dissolving prepackaged tablets in deionized water; one tablet in 200 mL of deionized water yields 0.01 M phosphate buffer, 0.0027 M potassium chloride, and 0.137 M sodium chloride at pH 7.4. An Ag/AgCl electrode in saturated 3 M NaCl solution was used as the reference electrode, and glassy carbon was used as the counter electrode for all electrochemical experiments. Cyclic voltammetry experiments were performed using 1 mM KI in 1X PBS and in synthetic urine. Electrochemical Impedance Spectroscopy (EIS) was performed after each cyclic voltammetry measurement to determine the active surface area of each electrode. Differential pulse voltammetry (DPV) experiments were run in both 1X PBS solution and synthetic urine with stepwise additions of KI, resulting in KI concentrations ranging from 100 nM to 1 mM.

## 3. Results/Discussion

### 3.1. Material Characterization

A set of materials characterizations, including Raman and XPS spectroscopies, and electron microscopy, was conducted to determine the chemical and structural properties of MCNF.

**Figure 2:**
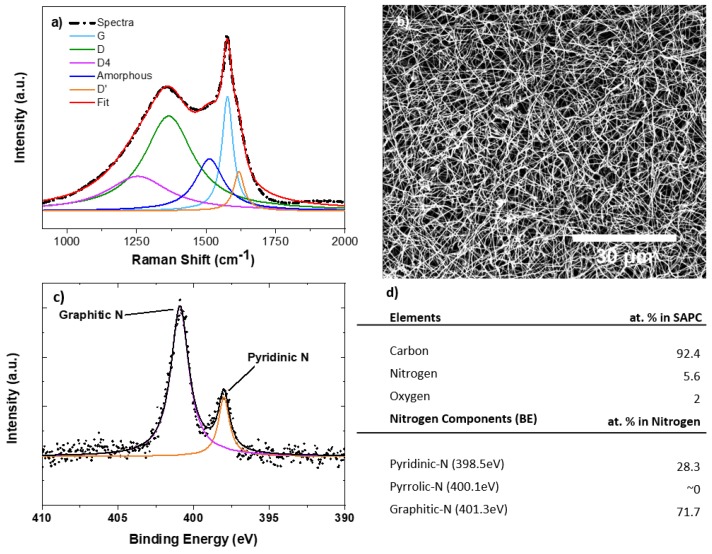
(**a**) Raman spectrum with fitted Lorentzian curves for MCNF electrodes. More than 100 Raman spectra collections (λ excitation = 532 nm) were averaged to analyze MCNF; (**b**) Scanning electron micrograph of MCNF’s; (**c**) XPS N 1s peak of MCNF; (**d**) Elemental composition of the carbon electrodes as analyzed from XPS.

In Figure 2b, the scanning electron micrographs of the resulting MCNF shows these nanofibers are relatively uniform and have an average diameter of 250 nm. The averaged Raman spectrum of the MCNF were obtained from 100 spectra of random areas on each carbon electrode to investigate the level of graphitization of the carbon materials. The D peak at 1350 cm^−1^, and G peak at 1600 cm^−1^ correlate with structural disorder and crystalline graphitic phase, respectively. Accordingly, the *I_d_*/*I_g_* peak intensity ratio—a standard criterion for assessing the quality of carbon—is determined to be 0.86, showing the graphitic quality to be higher compared to typical untreated PAN-based carbon nanofibers. However, as noted previously [14,15], graphitization alone is not enough to enhance the electrochemical performance of carbon materials. Instead, the presence of the right graphitic “disorders” and heteroatoms play a major role in the fast-electrochemical kinetics of carbon materials. As shown in our previous report [14], transmission electron microscopy (TEM) shows that the main source of disorders in MCNF are from carbon edge plane, which provide electroactive sites for efficient electron transfer. X-ray photoelectron spectroscopy (XPS) allows us to search for the presence of heteroatoms via analyzing various carbon bonds within the carbon structure (Figure 2c). The table in Figure 2d breaks down the elemental composition of MCNF, revealing a significant quantity of various nitrogen groups. These nitrogen groups consist of primarily pyridinic and graphitic, which have been associated with enhancing the electrocatalysis of graphitic carbons [15,16,17].

### 3.2. Electrochemical Characterization

#### 3.2.1. Cyclic Voltammetry

**Figure 3:**
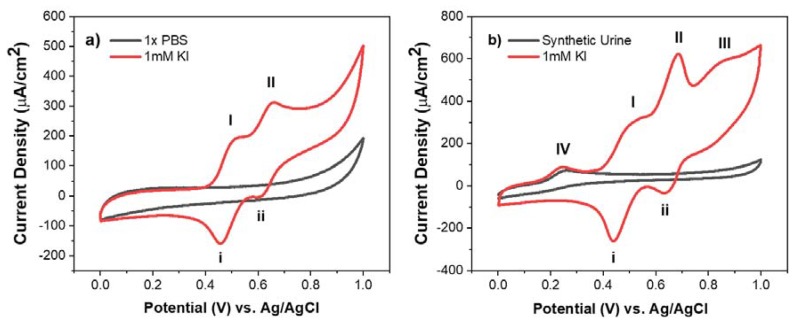
Cyclic voltammograms of 1 mM potassium iodide in (**a**) 1X PBS and (**b**) synthetic urine with a scan rate of 5 mV/s. The two-redox reactions of iodide ions are shown as (I)/(II) and (i)/(ii), respectively in both 1X PBS and synthetic urine. The additional peaks are observed in (**b**) attributed to the oxidation of unknown species occurring in the presence of potassium iodide (III) or the oxidation of creatinine in synthetic urine (IV).

The cyclic voltammogram (CV) shows the electrochemical response of MCNF electrodes in the presence of 1 mM potassium iodide in 1X PBS and in synthetic urine, summarized in Figure 3. In both media, the presence of redox peaks (I/i) and (II/ii) is attributed to a two-step redox reaction, summarized in the equations below:

(1) $$3I^{-}\Leftrightarrow I_{3}{}^{-}+2e^{-}$$

(2) $$2I_{3}{}^{-}\Leftrightarrow 3I_{2}+2e^{-}$$

Peak I shows the oxidation of iodide ions (*I*^−^) to triiodide (*I*_3_^−^) and peak II shows the oxidation of triiodide to iodine (*I*_2_). The redox potential ($E_{o\;})$ for a redox couple allows us to assess the kinetic behavior of an electrochemical reaction. For the iodide/triiodide redox reaction (I/i) in 1X PBS and in synthetic urine, the calculated $E_{o}$ values were 0.486 ± 0.004 V and 0.476 ± 0.002 V, respectively. This minor shift in the redox potentials shows that the redox reaction of (I/i) remains largely unaffected by a change in environment from 1X PBS to synthetic urine. This observation was corroborated by comparing the peak intensity of the reaction in both solvents, measured to be 165 ± 2 μA/${\mathrm{cm}}^{2}$ in 1x PBS and 170 ± 11 μA/${\mathrm{cm}}^{2}$, in synthetic urine. Similarly, very little shift in the $E_{o}$ values of the triiodide to iodine (peak II/ii) redox reaction was observed (0.636 ± 0.003 V and 0.639 ± 0.002 V in 1X PBS and synthetic urine, respectively). In this case, however, a 19% increase in current density was observed in synthetic urine (140 ± 5 μA/${\mathrm{cm}}^{2}$ in synthetic urine vs. 114 ± 2 μA/${\mathrm{cm}}^{2}$ in 1X PBS). In CV, the current density is directly related to the ionic flux, determined by the diffusion and transport of ions to the surface of the electrode. This increase in current density may be caused by the presence of heterogeneous chemical compounds that facilitated the transport of triiodide ions and promoted the overall oxidation of triiodide.

The additional peaks (III) and (IV) further confirm the presence of heterogeneous species in synthetic urine. In Figure 3b, peak (IV) was seen with and without the presence of 1 mM potassium iodide, indicating that the peak comes from endogenous chemical compounds that also participate in the overall redox system. Based on the work of Lopez-Giacoman et al., the observed peak indicates the oxidation of creatinine, an essential marker for diagnosing kidney-related disorders or muscular dystrophies [18]. Conversely, peak (III) was only observed when 1 mM KI was added to synthetic urine. This electrochemical phenomenon suggests that additional urinary species are being electrochemically oxidized in the presence of iodide ions. Further investigation of the exact oxidation process occurring at peak III is needed. Overall, based on CV results, MCNF electrodes have demonstrated effective selectivity towards iodide ions within complex biological media.

#### 3.2.2. Differential Pulse Voltammetry

Differential pulse voltammetry (DPV) is a highly sensitive pulse voltammetric technique that can discriminate against capacitive current in favor of oxidation or reduction currents, yielding sharp, well-defined peaks at lower concentrations of analyte. As a result, we were able to apply this technique to the quantitative analysis of iodide in both 1X PBS buffer and synthetic urine. DPV was run with a scan rate of 25 mV/s, a pulse height of 75 mV, and pulse width of 0.05 s. Analysis was performed by gradually increasing the concentration of KI in 1X PBS or synthetic urine and measuring the current density of corresponding peak (II) with respect to the background. The components of synthetic urine and their corresponding concentrations are listed in Table 1.

**Figure 4:**
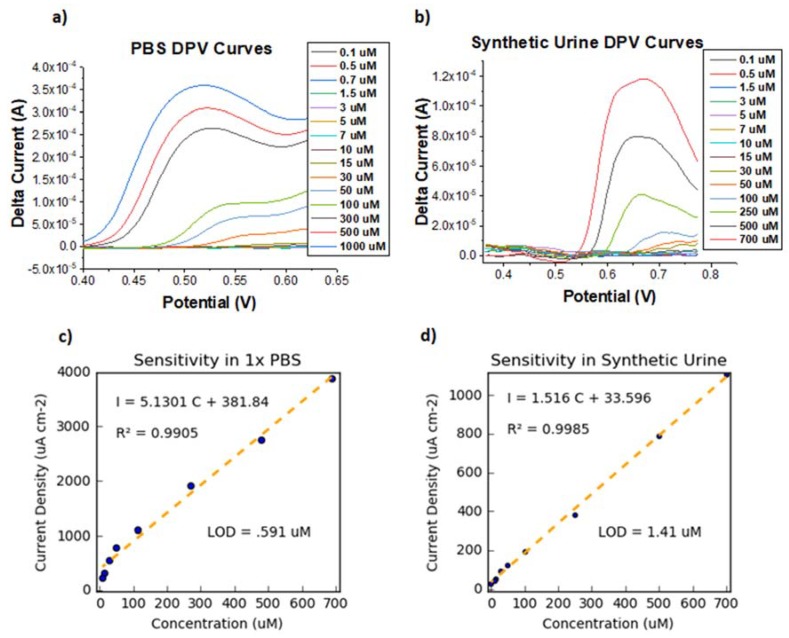
DPV curves (background subtracted) and calibration plots based on DPV data showing iodide sensitivity in 1X PBS (**a**,**c**) and synthetic urine (**b**,**d**). The regression equation, correlation coefficient, and estimated LOD are also shown on the plot.

The calibration plots in Figure 4 show a linear relationship between the peak height and concentration of iodide ions. Statistical analysis of the data corroborated this linearity by yielding high values of the correlation coefficient in the concentration range from 5 uM to 700 uM (R^2^ > 0.99 for both 1X PBS and synthetic urine). This linearity allows us to determine the limit of detection (LOD) and dynamic range of our MCNF. The LOD quantifies the lowest amount of analyte that can be detected using MCNF electrodes. LOD values were calculated according to the following equation [19]:

(3) $$LOD=\frac{3\;\sigma }{S}$$

where *σ* is the standard deviation of the data and *S* is the sensitivity, found by calculating the slope of the calibration curve. Using this equation, an *LOD* of 0.591 μM was estimated for MCNF in 1X PBS and an *LOD* of 1.41 μM in synthetic urine. For healthy adults, the WHO defines IDD as a urinary iodide concentration below 1.58 μM (<100 μg/L). The full spectrum of IDD is classified as mild deficiency, moderate deficiency <0.77 μM (<40 ug/L), and severe deficiency <0.32 μM (<20 ug/L). While an array of different sensors is required to fully determine the levels of IDD, our MCNF electrodes excel as rapid and inexpensive, qualitative sensors for IDD, particularly in complex urine samples.

The sensitivity of our MCNF electrode towards iodide ions in the presence of interfering ions was demonstrated by conducting DPV in synthetic urine. Table 1 shows the components of the synthetic urine used in the voltammetry experiments, with the principal interfering ions (i.e., urea and creatinine) present in concentrations similar to those of clinical urine specimens [20,21]. Figure 4b indicates that our MCNF electrode continues to demonstrate selectivity and sensitivity towards iodide ions in the presence of clinically appropriate concentrations of these interfering ions. The selectivity of our sensor for iodide ions is further supported by the CV’s obtained in synthetic urine. The results of the DPV and CV studies act as a proof of concept, demonstrating that our material not only remains sufficiently sensitive to iodide ions in complex media, but also differentiates among various interfering species in solution.

Moreover, the wide dynamic range of our sensor allows us to address additional iodine disorders occurring at excessive iodine concentrations. Excessive iodine, defined at concentrations greater than 4.73 μM (>300 μg/L), contributes to adverse health consequences such as hyperthyroidism and autoimmune thyroid disease [3]. These results, combined with the advantage of minimal sample preparation needed for testing, demonstrate the promising nature of MCNF sensors as an ideal point-of-care (POC) platform, particularly in rural areas with limited access to complex biomedical laboratories.

## 4. Conclusions

We investigated the capability of mechanically-treated carbon nanofibers (MCNF) as a promising platform for diagnosing both iodine deficiency (IDD) and iodine excess in urine. IDD is the most important preventable cause of neurological deficiencies worldwide and excess iodine can lead to thyroid-related disorders [2,6]. The unique microstructure and composition of MCNF electrodes make them uniquely suited for electrochemical sensing of iodine [14,15]. The cyclic voltammograms of MCNF electrodes in synthetic urine and PBS buffer demonstrate their efficacy in sensing and differentiating iodide ions from other interfering urinary species, without the need for complicated sample preparation and post-processing of the probe. Further quantitative analysis by DPV tests reveals that MCNF electrodes exhibit wide dynamic ranges that allow detection of excess iodine and limits of detection that allow for diagnosis of mild IDD. This work represents a proof of concept for using MCNF electrodes as POC sensors for iodine screening, particularly in low-resource setting areas.

## Figures and Tables

**Table 1:** Chemical composition of synthetic urine. The following table indicates the known concentration (g/L) of various components present in synthetic urine processed from Pickering Laboratories, Inc.

Component	Concentration (g/L)
Urea	25
Sodium Chloride	9
Disodium Hydrogen Orthophosphate, anhydrous	2.5
Potassium Dihydrogen Orthophosphate	2.5
Ammonium Chloride	3
Creatinine	2
Sodium Sulphite, hydrated	3
